# Supplementary figures and images for: Habitual coffee consumption poorly correlates with sleep quality and daytime sleepiness: A cross-sectional study
Source: PLoS One. 2026 Mar 9;21(3):e0344479. doi: 10.1371/journal.pone.0344479 (PMC12970861; doi:10.1371/journal.pone.0344479)

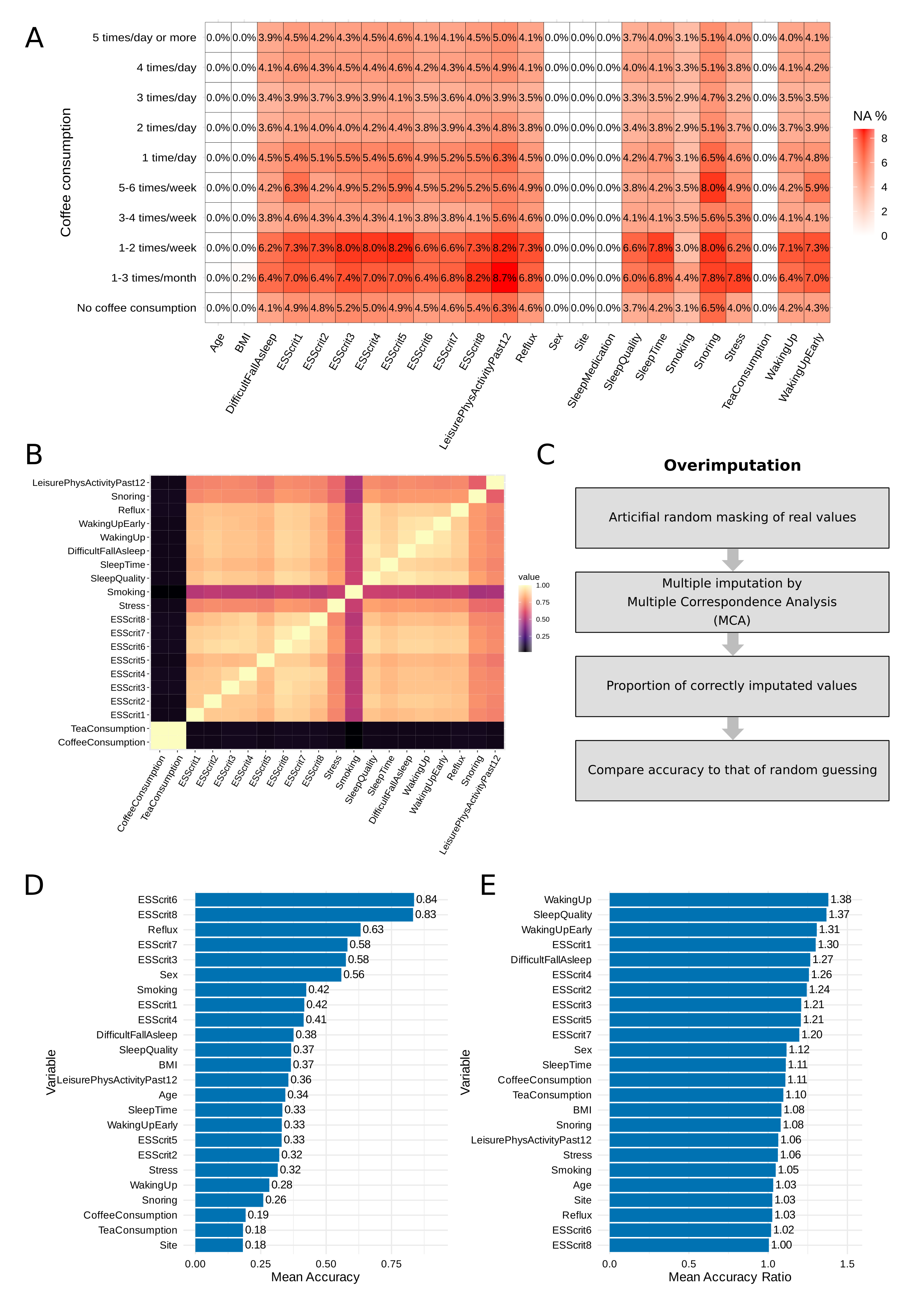

Supplement: S1 Fig — (A) Heatmap showing missingness patterns for each variable (x-axis) across coffee consumption levels (y-axis) before aggregation of coffee consumption into fewer groups. Values in each cell corresponds to percentage of missing values. (B) Heatmap showing Pearson correlation of missing values between all variables. The color map corresponds to the Pearson correlation coefficient. (C) Schematic overview of the procedure to assess the efficiency and accuracy of the data imputation, using an overimputation procedure. (D) Horizontal bar plot showing the mean accuracy of overimputed values. A value of 1 corresponds to perfect accuracy. (E) Horizontal bar plot showing the ratio between overimputation accuracy and random guessing. Values close to 1 indicate that that the imputation is not better than random guessing. (TIF) [file pone.0344479.s001.tif]

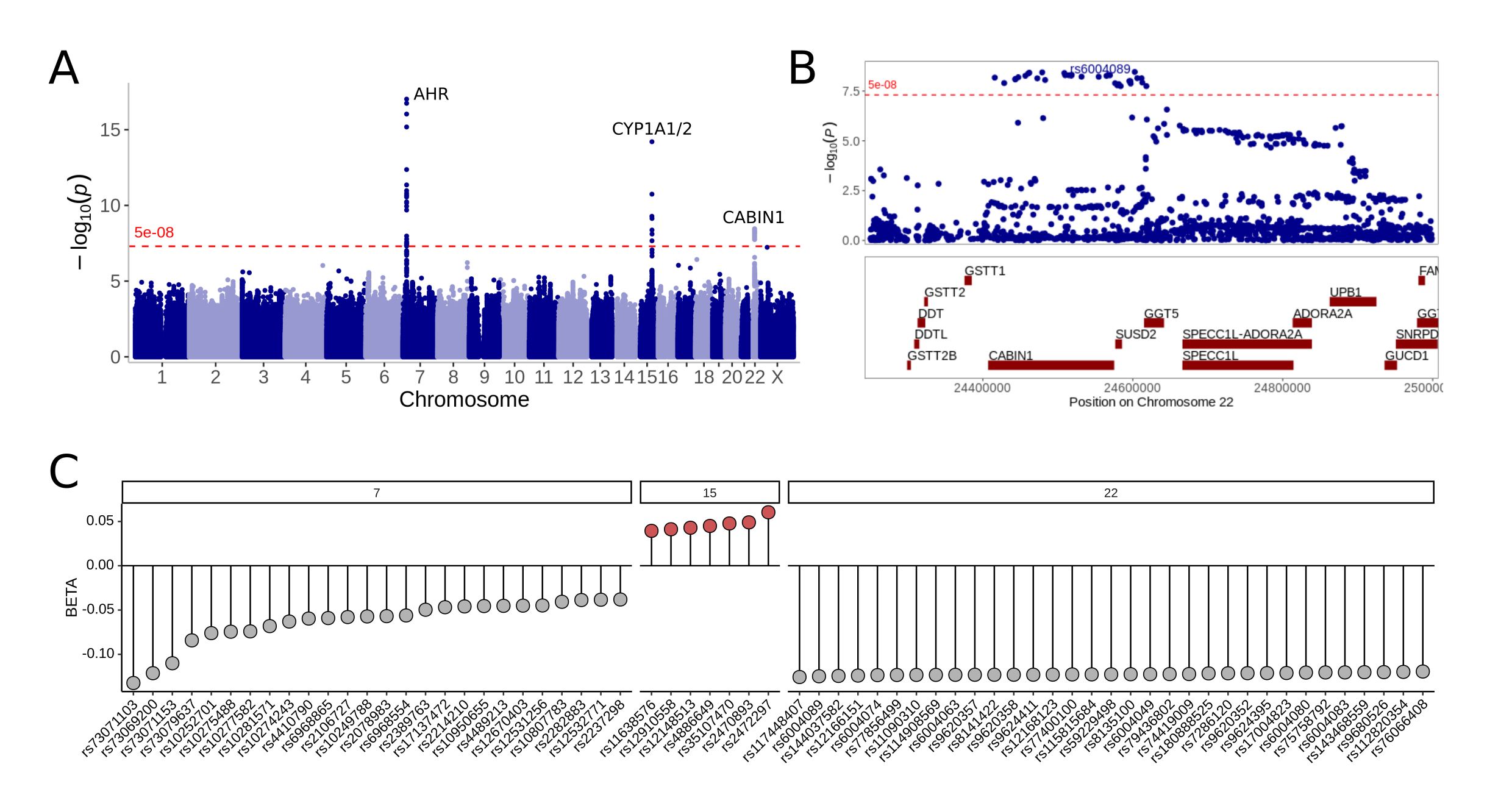

Supplement: S2 Fig — (A) Manhattan plot showing the p-values of the entire GWAS on a genomic scale, for coffee consumption. Blue points represent individual SNPs tested in the analysis. A p-value threshold of 5e-08 was used for determining significance (red-colored horizontal dashed line). (B) Region plot showing zoomed-in view at chromosome 22. Blue points represent individual SNPs. A p-value threshold of 5e-08 was used for determining significance (red-colored horizontal dashed line). Top significantly associated SNPs at each of the identified loci have been annotated in the plots. (C) Horizontal lollipop plot showing effect sizes (beta value) for each significant SNP. (TIF) [file pone.0344479.s002.tif]

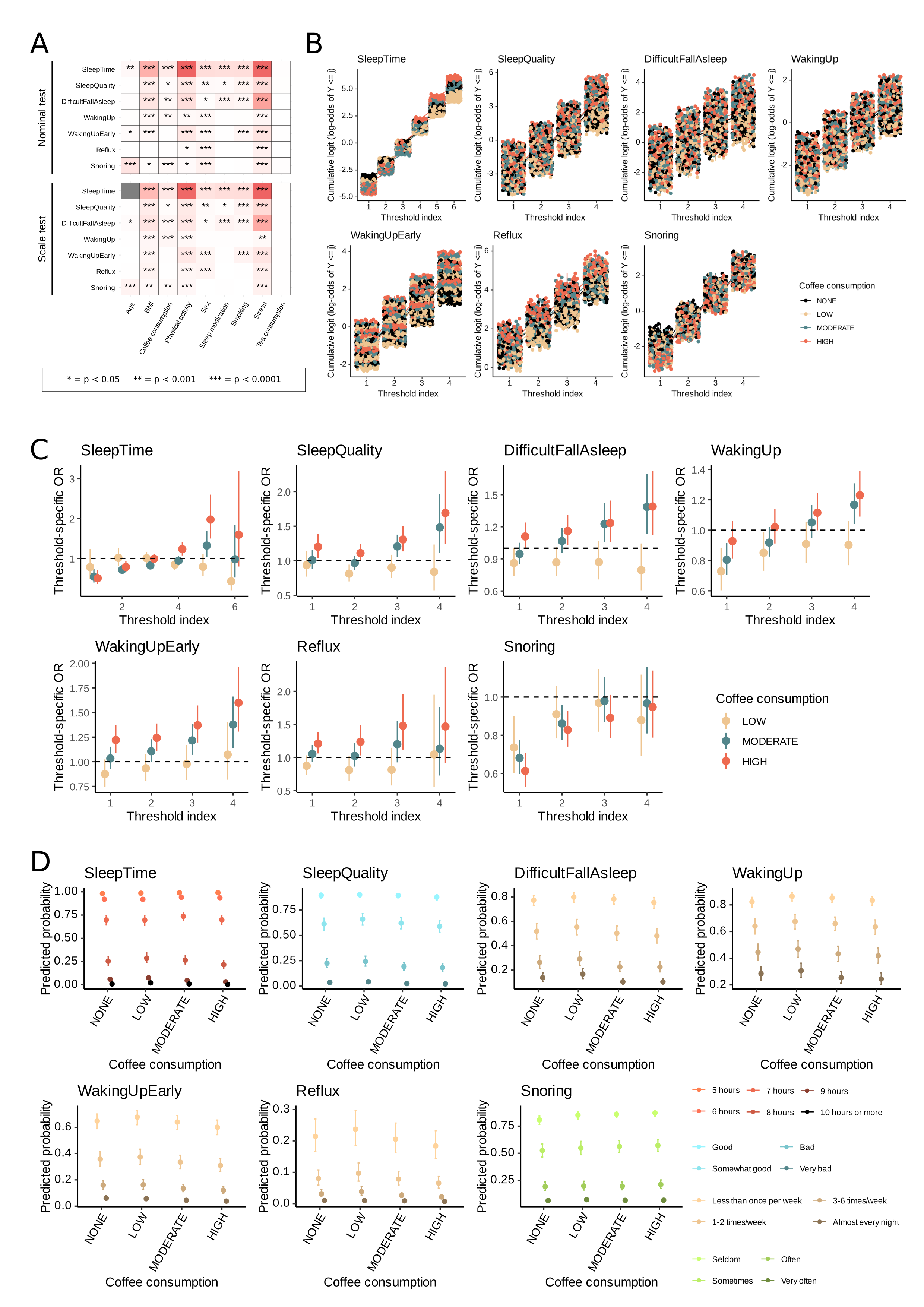

Supplement: S3 Fig — (A) Nominal test and scale test assessing the proportional odds assumption (PO). The heatmap shows the statistical significance obtained by these two tests for each of the modeled sleep traits (y-axis) and each predictor/covariate included in the models (x-axis). Color map corresponds to p-value. * = p < 0.05, ** = p < 0.001, *** = p < 0.0001. (B) Predicted cumulative logits for each coffee group plotted across the thresholds of each sleep variable. A roughly constant vertical separation between coffee groups across thresholds indicates that the PO holds in logit-space. In the present analysis, some changes in separation are observed, pointing to a possible violation of PO. (C) Threshold-specific odds ratios (ORs) obtained by fitting a partial proportional odds model. A large spread in ORs would indicate statistical non-proportionality. I the present analysis, ORs follow similar patterns. (D) Predicted category probabilities (y-axis) across coffee consumption groups (x-axis). Categories of each sleep variable are color-coded (see figure legend). Predicted-probability differences are small across categories, indicating that the PO violation is not practically important. (TIF) [file pone.0344479.s003.tif]

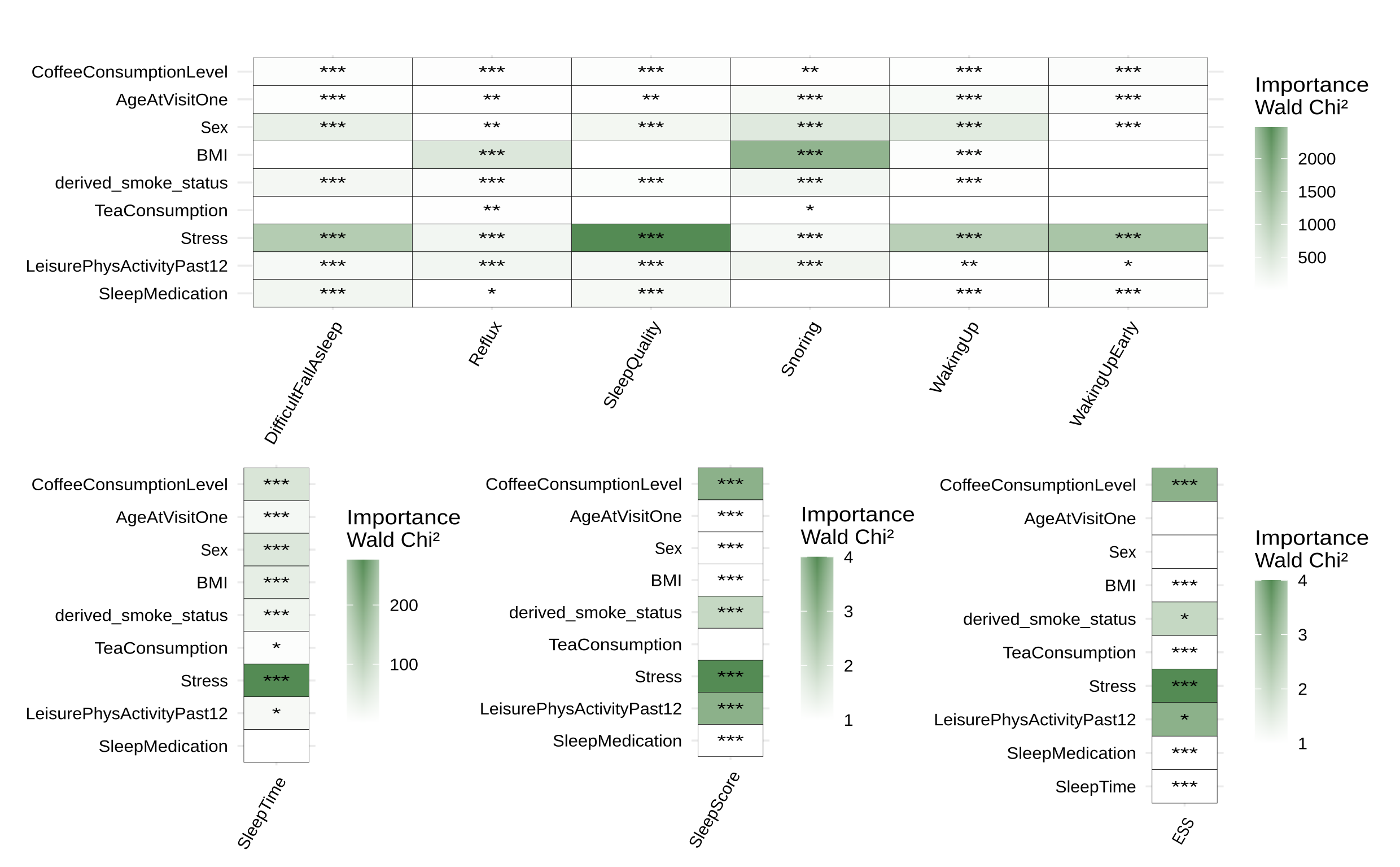

Supplement: S5 Fig — Variable importance of each factor (y-axis) for each fitted regression model (x-axis), assessed by analysis of variance (ANOVA). * = p < 0.05, ** = p < 0.001, *** = p < 0.0001. Color code represents the Type II Wald Chi-square statistics, where higher value (darker color) corresponds to a stronger contribution of the variable to the model. (TIF) [file pone.0344479.s005.tif]

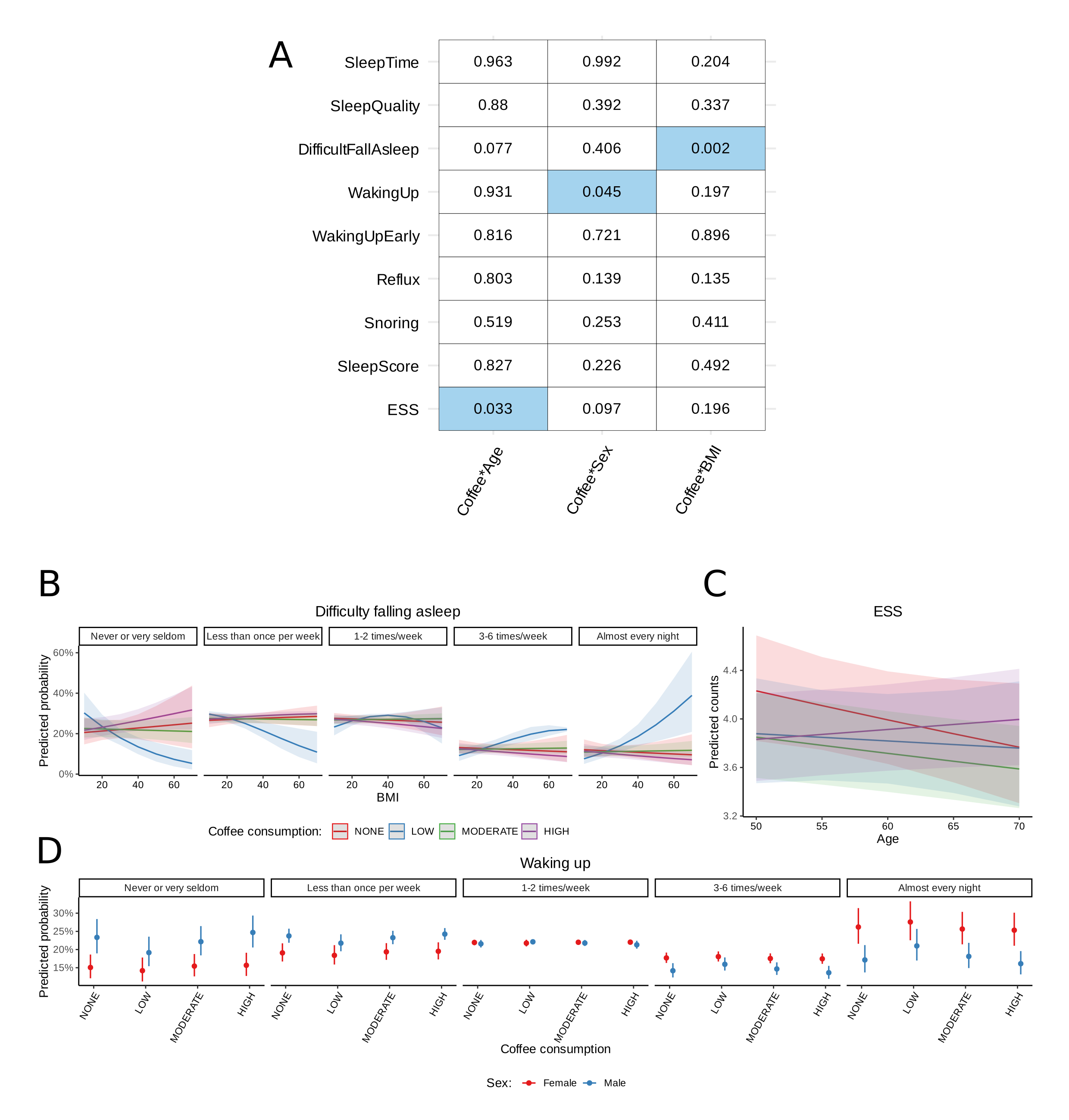

Supplement: S6 Fig — Investigation of potential effect modification by sex, age and BMI. (A) Assessment of improved model fit when introducing an interaction term between coffee consumption and age, sex and BMI, respectively (x-axis). This was performed for each of the outcomes studied (y-axis). For ordinal models, a likelihood ratio test was used. For quasi-Poisson models, an F-test was used. Significant results (p < 0.05) are highlighted in blue. (B) Adjusted predicted category probabilities (y-axis) showing the effect of coffee consumption level on difficulty to fall asleep, stratified by BMI (x-axis). (C) Adjusted predicted counts (y-axis) showing the effect of coffee consumption level on ESS score, stratified by age (x-axis). (D) Adjusted predicted category probabilities (y-axis) showing the effect of coffee consumption level (x-axis) on frequency waking up during night, stratified by sex. (TIF) [file pone.0344479.s006.tif]
