# Supplementary material for: Habitual coffee consumption poorly correlates with sleep quality and daytime sleepiness: A cross-sectional study
Source: PLoS One. 2026 Mar 9;21(3):e0344479. doi: 10.1371/journal.pone.0344479 (PMC12970861; doi:10.1371/journal.pone.0344479)
Supplement: S1 Table — Details on each SCAPIS variable selected for the present study, including method of data collection, questionnaire question wording and possible answers, and how the variable was used in the present study. (DOCX) [file pone.0344479.s007.docx]

***S1 Table. Details on each SCAPIS variable selected for the present study.*** *Details on each SCAPIS variable selected for the present study, including method of data collection, questionnaire question wording and possible answers, and how the variable was used in the present study.*

| Variable | Type | Details* | Usage in this study |
| --- | --- | --- | --- |
| Coffee consumption  Original variable label: q5question2 | Web-based food frequency questionnaire | Part of multiple-choice question.  Question wording [ENG]: “For each beverage you consume at least once a month, select from the drop-down list how often you typically consume it. Response selection: Coffee”  Possible answers:   - Not applicable - 5 times/day or more - 4 times/day - 3 times/day - 2 times/day - 1 time/day - 5-6 times/week - 3-4 times/week - 1-2 times/week - 1-3 times/month | Main predictor in all regression models.  Answers “Not applicable” was interpreted as “no coffee consumption. |
| Sleep duration  Original variable label: cqsh002 | Self-administered questionnaire on sleep habits | Question wording [ENG]: “How many hours do you normally sleep in a day?”  Possible answers:   - Don't want to/can't answer - 4 hours or less - 5 hours - 6 hours - 7 hours - 8 hours - 9 hours - 10 hours or more | Used as outcome variable in regression modelling.  Used in calculation of overall sleep score. |
| Sleep quality  Original variable label: cqsh001 | Self-administered questionnaire on sleep habits | Question wording [ENG]: “How do you usually sleep?”  Possible answers:   - Don't want to/can't answer - Very good - Good - Fairly good - Poor - Very poor | Used as outcome variable in regression modelling.  Used in calculation of overall sleep score. |
| Difficulty falling asleep  Original variable label: cqsh003 | Self-administered questionnaire on sleep habits | Question wording [ENG]: “How often do you have trouble falling asleep at night?”  Possible answers:   - Don't want to/can't answer - Never/almost never - Less than once a week - 1-2 times a week - 3-6 times a week - Almost every night | Used as outcome variable in regression modelling.  Used in calculation of overall sleep score. |
| Frequency waking up at night  Original variable label: cqsh004 | Self-administered questionnaire on sleep habits | Question wording [ENG]: “How often do you wake up repeatedly during the night?”  Possible answers:   - Don't want to/can't answer - Never/almost never - Less than once a week - 1-2 times a week - 3-6 times a week - Almost every night | Used as outcome variable in regression modelling.  Used in calculation of overall sleep score. |
| Frequency waking up too early  Original variable label: cqsh005 | Self-administered questionnaire on sleep habits | Question wording [ENG]: “How often do you wake up too early and can't get back to sleep?”  Possible answers:   - Don't want to/can't answer - Never/almost never - Less than once a week - 1-2 times a week - 3-6 times a week - Almost every night | Used as outcome variable in regression modelling.  Used in calculation of overall sleep score. |
| Reflux  Original variable label: cqsh014 | Self-administered questionnaire on sleep habits | Question wording [ENG]: “How often do you experience heartburn or acid reflux after going to bed?”  Possible answers:   - Don't want to/can't answer - Never/almost never - Less than once a week - 1-2 times a week - 3-6 times a week - Almost every night | Used as outcome variable in regression modelling.  Used in calculation of overall sleep score. |
| Snoring  Original variable label: cqsh015 | Self-administered questionnaire on sleep habits | Question wording [ENG]: “Do you snore loudly (according to yourself or others)?”  Possible answers:   - Don't want to/can't answer - Never - Rarely - Sometimes - Often - Very often | Used as outcome variable in regression modelling.  Used in calculation of overall sleep score. |
| Chance of dozing: sitting and reading  Original variable label: cqsh006 | Self-administered questionnaire on sleep habits | Part of the 8 Epworth Sleepiness Scale (ESS) questions.  Question wording [ENG]: “How likely are you to doze off or fall asleep in the following situations, in contrast to feeling just tired? This refers to your usual way of life in recent times. Even if you haven't done some of these things recently, try to work out how they would have affected you. Response alternative: Sitting and reading”  Possible answers:   - Cannot/will not answer - None - Small - Moderate - Large | Used in calculation of overall ESS score. |
| Chance of dozing: watching TV  Original variable label: cqsh007 | Self-administered questionnaire on sleep habits | Part of the 8 Epworth Sleepiness Scale (ESS) questions.  Question wording [ENG]: “How likely are you to doze off or fall asleep in the following situations, in contrast to feeling just tired? This refers to your usual way of life in recent times. Even if you haven't done some of these things recently, try to work out how they would have affected you. Response alternative: Watching TV”  Possible answers (also applicable to the next seven scenarios on chance of dozing):   - Cannot/will not answer - None - Small - Moderate - Large | Used in calculation of overall ESS score. |
| Chance of dozing: sitting inactive in a public place (e.g. theatre or a meeting)  Original variable label: cqsh008 | Self-administered questionnaire on sleep habits | Part of the 8 Epworth Sleepiness Scale (ESS) questions.  Question wording [ENG]: “How likely are you to doze off or fall asleep in the following situations, in contrast to feeling just tired? This refers to your usual way of life in recent times. Even if you haven't done some of these things recently, try to work out how they would have affected you. Response alternative: Sitting, inactive in a public place (e.g. a theatre or a  meeting)” | Used in calculation of overall ESS score. |
| Chance of dozing: as a passenger in a car for an hour without a break  Original variable label: cqsh009 | Self-administered questionnaire on sleep habits | Part of the 8 Epworth Sleepiness Scale (ESS) questions.  Question wording [ENG]: “How likely are you to doze off or fall asleep in the following situations, in contrast to feeling just tired? This refers to your usual way of life in recent times. Even if you haven't done some of these things recently, try to work out how they would have affected you. Response alternative: As a passenger in a car for an hour without break” | Used in calculation of overall ESS score. |
| Chance of dozing: lying down to rest in the afternoon when circumstances permit  Original variable label: cqsh010 | Self-administered questionnaire on sleep habits | Part of the 8 Epworth Sleepiness Scale (ESS) questions.  Question wording [ENG]: “How likely are you to doze off or fall asleep in the following situations, in contrast to feeling just tired? This refers to your usual way of life in recent times. Even if you haven't done some of these things recently, try to work out how they would have affected you. Response alternative: Lying down to rest in the afternoon when circumstances permit” | Used in calculation of overall ESS score. |
| Chance of dozing: sitting and talking to someone  Original variable label: cqsh011 | Self-administered questionnaire on sleep habits | Part of the 8 Epworth Sleepiness Scale (ESS) questions.  Question wording [ENG]: “How likely are you to doze off or fall asleep in the following situations, in contrast to feeling just tired? This refers to your usual way of life in recent times. Even if you haven't done some of these things recently, try to work out how they would have affected you. Response alternative: Sitting and talking to someone” | Used in calculation of overall ESS score. |
| Chance of dozing: sitting quietly after a lunch without alcohol  Original variable label: cqsh012 | Self-administered questionnaire on sleep habits | Part of the 8 Epworth Sleepiness Scale (ESS) questions.  Question wording [ENG]: “How likely are you to doze off or fall asleep in the following situations, in contrast to feeling just tired? This refers to your usual way of life in recent times. Even if you haven't done some of these things recently, try to work out how they would have affected you. Response alternative: Sitting quietly after a lunch without alcohol” | Used in calculation of overall ESS score. |
| Chance of dozing: in a car, while stopped for a few minutes in the traffic  Original variable label: cqsh013 | Self-administered questionnaire on sleep habits | Part of the 8 Epworth Sleepiness Scale (ESS) questions.  Question wording [ENG]: “How likely are you to doze off or fall asleep in the following situations, in contrast to feeling just tired? This refers to your usual way of life in recent times. Even if you haven't done some of these things recently, try to work out how they would have affected you. Response alternative: In a car, while stopped for a few minutes in the traffic” | Used in calculation of overall ESS score. |
| Age  Original variable label: AgeAtVisitOne | Demography (register data) | Age at study visit 1, rounded to 1 decimal. The decimal digit denotes the decimal fraction of a whole year (i.e. it does not denote month number). | Included as covariate in regression analyses  Included as covariate in GWAS |
| Sex | Demography (register data) | Female  Male | Included as covariate in regression analyses  Included as covariate in GWAS |
| Site of examination | Demography (register data) | 1 – Gothenburg  2 – Malmö  3 – Stockholm  4 – Linköping  5 – Uppsala  6 – Umeå | Included as covariate in GWAS |
| Smoking  Original variable label: derived_smoke_status | Questionnaire | CURRENT  EX_SMOKER  NEVER  UNKNOWN | Included as covariate in regression analyses |
| Tea consumption  Original variable label: q5question3 | Web-based food frequency questionnaire | Part of multiple choice question.  Question wording [ENG]: “For each beverage you consume at least once a month, select from the drop-down list how often you typically consume it. Response selection: Tea”  Possible answers:   - Not applicable - 5 times/day or more - 4 times/day - 3 times/day - 2 times/day - 1 time/day - 5-6 times/week - 3-4 times/week - 1-2 times/week - 1-3 times/month | Included as covariate in regression analyses |
| Stress  Original variable label: q5question3 | Questionnaire | Question wording [ENG]: “By stress we mean feeling tense, irritable, anxious or having sleeping difficulties as a result of conditions at work or at home. Did you experience this?”  Possible answers:   - Don't want to/can't answer - Never experienced stress - Experienced some stress - Some stress in the last five years - Constant stress in the last year - Constant stress in the last five years | Included as covariate in regression analyses |
| Physical activity  Original variable label: cqpa012 | Questionnaire | Question wording [ENG]: “How much physical movement and exertion have you had in the last 12 months?”  Possible answers:   - Don't want to/can't answer - Sedentary leisure time. (You mostly spend your free time reading, watching TV, using computers, or doing other sedentary activities. You walk, cycle or exercise in other ways for less than 2 hours per week.) - Moderate exercise during leisure time. (You walk, cycle or engage in other forms of exercise for at least 4 hours per week. This includes, for example, walking or cycling to and from work, other walks, heavy housework, ordinary gardening, fishing, table tennis, bowling) - Moderate but regular exercise in your free time. You engage in, for example running, swimming, tennis, badminton, exercise classes, or similar activities. Heavy gardening and similar activities are included in this group. Please note that this should be an average of at least 2-3 hours per week. (You exercise regularly 1-2 times per week for at least 30 minutes per session with, for example, running, swimming, tennis, badminton, or other activities that make you sweat.) - Regular exercise and training. (You engage in, for example, strenuous training or competition in running, orienteering, skiing, swimming, soccer, handball, etc. regularly and several times a week. Tennis, badminton, gymnastics, or similar activities at least 3 times per week on average. Each session lasts at least 30 minutes per session.) | Included as covariate in regression analyses |
| Medications used  Original variable label: cqme006 | Questionnaire | Question wording [ENG]: “Write down the names of your medications below”  Free text. Answers as given by the subjects, including misspellings etc. No ATC coding is available. | Used to classify participants as sleep medication users (if they have reported use of any of the following: melatonin, mirtazapine, propavan, zolpidem, circadin, imovane or zopiklon).  The above classification “sleep medication user” (Yes/No), was included as covariate in regression analyses |
