# Supplementary material for: Habitual coffee consumption poorly correlates with sleep quality and daytime sleepiness: A cross-sectional study
Source: PLoS One. 2026 Mar 9;21(3):e0344479. doi: 10.1371/journal.pone.0344479 (PMC12970861; doi:10.1371/journal.pone.0344479)
Supplement: S2 Table — Columns from left to right: name of the SNP, the chromosome on which the SNP is located, the single nucleotide position of the SNP, the allele that was tested in the analysis, the alternative allele, mean allele frequency of the tested allele, the effect size estimate (beta) of the SNP, the standard error (se) of the effect size estimate, and the statistical significance (p-value) for the association. (DOCX) [file pone.0344479.s008.docx]

***S2 Table.*** ***Significant associated SNPs obtained by GWAS on coffee consumption.*** *Columns from left to right: name of the SNP, the chromosome on which the SNP is located, the single nucleotide position of the SNP, the allele that was tested in the analysis, the alternative allele, mean allele frequency of the tested allele, the effect size estimate (beta) of the SNP, the standard error (se) of the effect size estimate, and the statistical significance (p-value) for the association.*

| SNP name | Chr. | Position | Effect allele | Other allele | MAF | β | σ_β_ | P-value |
| --- | --- | --- | --- | --- | --- | --- | --- | --- |
| rs10252701 | 7 | 17280513 | C | A | 0.0984173 | -0.0760534 | 0.0116086 | 5.79E-11 |
| rs12531256 | 7 | 17281820 | C | T | 0.467269 | -0.0448465 | 0.00673331 | 2.78E-11 |
| rs4410790 | 7 | 17284577 | T | C | 0.360507 | -0.0596339 | 0.00694626 | 9.53E-18 |
| rs10277582 | 7 | 17284989 | T | C | 0.0985515 | -0.0740874 | 0.0114855 | 1.13E-10 |
| rs10281571 | 7 | 17285867 | G | C | 0.117383 | -0.0681563 | 0.0107142 | 2.03E-10 |
| rs6968554 | 7 | 17287106 | A | G | 0.358818 | -0.0561163 | 0.00694304 | 6.60E-16 |
| rs6968865 | 7 | 17287269 | A | T | 0.36157 | -0.0590804 | 0.00694169 | 1.81E-17 |
| rs2106727 | 7 | 17287998 | A | G | 0.357294 | -0.0579179 | 0.00696189 | 9.24E-17 |
| rs17137472 | 7 | 17296072 | C | T | 0.467389 | -0.0468186 | 0.00676014 | 4.43E-12 |
| rs10275488 | 7 | 17303778 | T | C | 0.0997517 | -0.0744321 | 0.0113996 | 6.72E-11 |
| rs73079637 | 7 | 17306897 | C | T | 0.0559782 | -0.0842541 | 0.0151602 | 2.76E-08 |
| rs2214210 | 7 | 17307374 | G | A | 0.467209 | -0.0459246 | 0.00675323 | 1.06E-11 |
| rs2389763 | 7 | 17307847 | T | C | 0.40173 | -0.0496579 | 0.00685009 | 4.30E-13 |
| rs10950655 | 7 | 17308367 | T | C | 0.463365 | -0.0455143 | 0.00675033 | 1.59E-11 |
| rs4489213 | 7 | 17309180 | G | A | 0.46832 | -0.0452614 | 0.00674666 | 2.00E-11 |
| rs12670403 | 7 | 17309279 | C | A | 0.468907 | -0.0450315 | 0.00673501 | 2.33E-11 |
| rs10807783 | 7 | 17313726 | T | G | 0.339673 | -0.0406978 | 0.00711104 | 1.06E-08 |
| rs10249788 | 7 | 17338147 | T | C | 0.116343 | -0.0572164 | 0.0104549 | 4.47E-08 |
| rs12532771 | 7 | 17341796 | A | G | 0.352146 | -0.0383512 | 0.00699615 | 4.25E-08 |
| rs2237298 | 7 | 17348119 | T | C | 0.358177 | -0.0380943 | 0.00697983 | 4.86E-08 |
| rs2282883 | 7 | 17356347 | T | C | 0.354576 | -0.0387091 | 0.00700555 | 3.31E-08 |
| rs2078983 | 7 | 17392620 | A | C | 0.122047 | -0.0569052 | 0.0103309 | 3.65E-08 |
| rs10274243 | 7 | 17403696 | A | G | 0.0960057 | -0.06297 | 0.0115209 | 4.65E-08 |
| rs73069200 | 7 | 17519844 | C | G | 0.0272096 | -0.121296 | 0.021498 | 1.69E-08 |
| rs73071103 | 7 | 17520240 | G | T | 0.0222573 | -0.13237 | 0.0239485 | 3.28E-08 |
| rs73071153 | 7 | 17545964 | A | G | 0.032053 | -0.110079 | 0.0197662 | 2.58E-08 |
| rs35107470 | 15 | 74817689 | G | A | 0.297019 | 0.0477514 | 0.00768404 | 5.22E-10 |
| rs2470893 | 15 | 75019449 | T | C | 0.304853 | 0.0489896 | 0.00728647 | 1.81E-11 |
| rs2472297 | 15 | 75027880 | T | C | 0.253397 | 0.0603772 | 0.0077372 | 6.23E-15 |
| rs12148513 | 15 | 75222225 | T | C | 0.347041 | 0.0428879 | 0.0072987 | 4.25E-09 |
| rs11638576 | 15 | 75260387 | A | G | 0.390334 | 0.0393857 | 0.00703363 | 2.17E-08 |
| rs4886649 | 15 | 75328595 | C | T | 0.335565 | 0.0449409 | 0.00729356 | 7.29E-10 |
| rs12910558 | 15 | 75396809 | T | C | 0.358082 | 0.0411757 | 0.00713025 | 7.78E-09 |
| rs144037582 | 22 | 24415916 | A | G | 0.026148 | -0.124385 | 0.0214463 | 6.71E-09 |
| rs9620352 | 22 | 24428449 | C | A | 0.0262699 | -0.121289 | 0.0213069 | 1.26E-08 |
| rs77400100 | 22 | 24443319 | A | G | 0.0262133 | -0.122702 | 0.0212874 | 8.30E-09 |
| rs12168123 | 22 | 24443361 | A | C | 0.0262429 | -0.122935 | 0.0212934 | 7.85E-09 |
| rs17004823 | 22 | 24447303 | A | G | 0.0265294 | -0.1211 | 0.020857 | 6.46E-09 |
| rs7286120 | 22 | 24456708 | C | T | 0.0267323 | -0.12141 | 0.0208073 | 5.44E-09 |
| rs9624395 | 22 | 24460593 | C | A | 0.0272262 | -0.121196 | 0.0206141 | 4.17E-09 |
| rs6004049 | 22 | 24461942 | A | G | 0.0268273 | -0.122416 | 0.0207713 | 3.82E-09 |
| rs79436802 | 22 | 24472596 | A | G | 0.026107 | -0.122309 | 0.0211405 | 7.30E-09 |
| rs12166151 | 22 | 24479193 | G | C | 0.0264789 | -0.123993 | 0.021016 | 3.68E-09 |
| rs8135100 | 22 | 24483100 | A | T | 0.0258927 | -0.122485 | 0.0212889 | 8.83E-09 |
| rs117448407 | 22 | 24508729 | A | G | 0.0258849 | -0.125634 | 0.0213385 | 3.96E-09 |
| rs9620357 | 22 | 24509835 | T | C | 0.0264432 | -0.123075 | 0.0210556 | 5.11E-09 |
| rs8141422 | 22 | 24514736 | A | C | 0.0264684 | -0.12307 | 0.0210563 | 5.13E-09 |
| rs9620358 | 22 | 24518086 | G | A | 0.0264354 | -0.123042 | 0.0210727 | 5.31E-09 |
| rs6004063 | 22 | 24518496 | A | G | 0.0265538 | -0.123144 | 0.0210328 | 4.83E-09 |
| rs59229498 | 22 | 24531662 | A | C | 0.0264728 | -0.12261 | 0.0210769 | 6.05E-09 |
| rs77856499 | 22 | 24554474 | T | A | 0.0262838 | -0.123328 | 0.0212057 | 6.10E-09 |
| rs9624411 | 22 | 24564915 | C | A | 0.0264893 | -0.123027 | 0.0210904 | 5.49E-09 |
| rs6004074 | 22 | 24567608 | T | C | 0.026478 | -0.123496 | 0.0210983 | 4.87E-09 |
| rs11090310 | 22 | 24569468 | T | C | 0.0265155 | -0.123252 | 0.0210882 | 5.13E-09 |
| rs180888525 | 22 | 24576371 | G | A | 0.0259328 | -0.121557 | 0.021364 | 1.28E-08 |
| rs9680526 | 22 | 24579503 | A | T | 0.0260669 | -0.120111 | 0.0212691 | 1.65E-08 |
| rs6004080 | 22 | 24581182 | C | T | 0.0261131 | -0.1206 | 0.0212584 | 1.42E-08 |
| rs112820354 | 22 | 24583829 | A | G | 0.0261401 | -0.119827 | 0.0212728 | 1.79E-08 |
| rs6004083 | 22 | 24595365 | G | T | 0.026836 | -0.120405 | 0.0210059 | 1.00E-08 |
| rs75758792 | 22 | 24596835 | G | C | 0.0268186 | -0.120451 | 0.0210181 | 1.01E-08 |
| rs76066408 | 22 | 24597154 | A | G | 0.0268247 | -0.119418 | 0.0210126 | 1.34E-08 |
| rs6004089 | 22 | 24602592 | C | T | 0.0266627 | -0.124766 | 0.0211142 | 3.48E-09 |
| rs74419009 | 22 | 24606001 | G | T | 0.0264989 | -0.12227 | 0.0211216 | 7.16E-09 |
| rs114908569 | 22 | 24611690 | C | T | 0.0263517 | -0.123212 | 0.0212874 | 7.20E-09 |
| rs115815684 | 22 | 24612679 | A | G | 0.025965 | -0.122657 | 0.021503 | 1.18E-08 |
| rs143468559 | 22 | 24618103 | T | C | 0.0266017 | -0.120175 | 0.0213368 | 1.80E-08 |
