# Supplementary material for: Habitual coffee consumption poorly correlates with sleep quality and daytime sleepiness: A cross-sectional study
Source: PLoS One. 2026 Mar 9;21(3):e0344479. doi: 10.1371/journal.pone.0344479 (PMC12970861; doi:10.1371/journal.pone.0344479)
Supplement: S3 Table — (DOCX) [file pone.0344479.s009.docx]

| ***S3 Table.*** *Patterns of habitual coffee consumption by sleep factors and ESS.* | | | | | | |
| --- | --- | --- | --- | --- | --- | --- |
|  |  | **Coffee consumption** | | | |  |
| **Characteristic** | **Overall** N = 25,381*^1^* | **NONE** N = 1,780*^1^* | **LOW** N = 1,344*^1^* | **MODERATE** N = 14,124*^1^* | **HIGH** N = 8,133*^1^* | **p-value***^2^* |
| **Hours of sleep** |  |  |  |  |  | <0.001 |
| 4 hours or less | 408 (1.6%) | 50 (2.8%) | 30 (2.2%) | 210 (1.5%) | 118 (1.5%) |  |
| 5 hours | 1,860 (7.3%) | 153 (8.6%) | 127 (9.4%) | 943 (6.7%) | 637 (7.8%) |  |
| 6 hours | 6,836 (27%) | 486 (27%) | 368 (27%) | 3,575 (25%) | 2,407 (30%) |  |
| 7 hours | 11,389 (45%) | 747 (42%) | 524 (39%) | 6,453 (46%) | 3,665 (45%) |  |
| 8 hours | 4,078 (16%) | 267 (15%) | 223 (17%) | 2,461 (17%) | 1,127 (14%) |  |
| 9 hours | 657 (2.6%) | 66 (3.7%) | 53 (3.9%) | 390 (2.8%) | 148 (1.8%) |  |
| 10 hours or more | 153 (0.6%) | 11 (0.6%) | 19 (1.4%) | 92 (0.7%) | 31 (0.4%) |  |
| **Quality of sleep** |  |  |  |  |  | <0.001 |
| Very good | 4,506 (18%) | 294 (17%) | 222 (17%) | 2,361 (17%) | 1,629 (20%) |  |
| Good | 8,018 (32%) | 566 (32%) | 386 (29%) | 4,420 (31%) | 2,646 (33%) |  |
| Somewhat good | 8,812 (35%) | 580 (33%) | 475 (35%) | 5,063 (36%) | 2,694 (33%) |  |
| Bad | 3,468 (14%) | 279 (16%) | 211 (16%) | 1,962 (14%) | 1,016 (12%) |  |
| Very bad | 577 (2.3%) | 61 (3.4%) | 50 (3.7%) | 318 (2.3%) | 148 (1.8%) |  |
| **Frequency of difficulty falling asleep** |  |  |  |  |  | <0.001 |
| Never or very seldom | 11,154 (44%) | 773 (43%) | 558 (42%) | 5,956 (42%) | 3,867 (48%) |  |
| Less than once per week | 6,974 (27%) | 463 (26%) | 354 (26%) | 4,027 (29%) | 2,130 (26%) |  |
| 1-2 times/week | 4,335 (17%) | 303 (17%) | 237 (18%) | 2,523 (18%) | 1,272 (16%) |  |
| 3-6 times/week | 1,587 (6.3%) | 119 (6.7%) | 87 (6.5%) | 899 (6.4%) | 482 (5.9%) |  |
| Almost every night | 1,331 (5.2%) | 122 (6.9%) | 108 (8.0%) | 719 (5.1%) | 382 (4.7%) |  |
| **Frequency waking up several times** |  |  |  |  |  | <0.001 |
| Never or very seldom | 4,532 (18%) | 351 (20%) | 215 (16%) | 2,328 (16%) | 1,638 (20%) |  |
| Less than once per week | 5,051 (20%) | 327 (18%) | 267 (20%) | 2,766 (20%) | 1,691 (21%) |  |
| 1-2 times/week | 5,314 (21%) | 329 (18%) | 267 (20%) | 3,045 (22%) | 1,673 (21%) |  |
| 3-6 times/week | 4,144 (16%) | 269 (15%) | 202 (15%) | 2,385 (17%) | 1,288 (16%) |  |
| Almost every night | 6,340 (25%) | 504 (28%) | 393 (29%) | 3,600 (25%) | 1,843 (23%) |  |
| **Frequency waking up too early** |  |  |  |  |  | <0.001 |
| Never or very seldom | 8,601 (34%) | 565 (32%) | 400 (30%) | 4,608 (33%) | 3,028 (37%) |  |
| Less than once per week | 7,319 (29%) | 487 (27%) | 389 (29%) | 4,143 (29%) | 2,300 (28%) |  |
| 1-2 times/week | 5,314 (21%) | 374 (21%) | 295 (22%) | 3,024 (21%) | 1,621 (20%) |  |
| 3-6 times/week | 2,699 (11%) | 212 (12%) | 164 (12%) | 1,532 (11%) | 791 (9.7%) |  |
| Almost every night | 1,448 (5.7%) | 142 (8.0%) | 96 (7.1%) | 817 (5.8%) | 393 (4.8%) |  |
| **Frequency of reflux after going to bed** |  |  |  |  |  | <0.001 |
| Never or very seldom | 19,681 (78%) | 1,348 (76%) | 965 (72%) | 10,929 (77%) | 6,439 (79%) |  |
| Less than once per week | 3,438 (14%) | 259 (15%) | 211 (16%) | 1,912 (14%) | 1,056 (13%) |  |
| 1-2 times/week | 1,466 (5.8%) | 103 (5.8%) | 99 (7.4%) | 840 (5.9%) | 424 (5.2%) |  |
| 3-6 times/week | 532 (2.1%) | 47 (2.6%) | 51 (3.8%) | 290 (2.1%) | 144 (1.8%) |  |
| Almost every night | 264 (1.0%) | 23 (1.3%) | 18 (1.3%) | 153 (1.1%) | 70 (0.9%) |  |
| **Frequency of loud snoring** |  |  |  |  |  | <0.001 |
| Never | 3,292 (13%) | 349 (20%) | 179 (13%) | 1,899 (13%) | 865 (11%) |  |
| Seldom | 6,548 (26%) | 452 (25%) | 344 (26%) | 3,730 (26%) | 2,022 (25%) |  |
| Sometimes | 8,818 (35%) | 559 (31%) | 451 (34%) | 4,960 (35%) | 2,848 (35%) |  |
| Often | 4,202 (17%) | 264 (15%) | 214 (16%) | 2,205 (16%) | 1,519 (19%) |  |
| Very often | 2,521 (9.9%) | 156 (8.8%) | 156 (12%) | 1,330 (9.4%) | 879 (11%) |  |
| **ESS category** |  |  |  |  |  | <0.001 |
| Lower Normal DS | 12,297 (48%) | 799 (45%) | 648 (48%) | 6,998 (50%) | 3,852 (47%) |  |
| Higher Normal DS | 9,014 (36%) | 602 (34%) | 455 (34%) | 5,025 (36%) | 2,932 (36%) |  |
| Mild Excessive DS | 1,887 (7.4%) | 148 (8.3%) | 101 (7.5%) | 984 (7.0%) | 654 (8.0%) |  |
| Moderate Excessive DS | 1,470 (5.8%) | 150 (8.4%) | 89 (6.6%) | 778 (5.5%) | 453 (5.6%) |  |
| Severe Excessive DS | 713 (2.8%) | 81 (4.6%) | 51 (3.8%) | 339 (2.4%) | 242 (3.0%) |  |
| *^1^*n (%) | | | | | | |
| *^2^*Pearson's Chi-squared test | | | | | | |
