# Supplementary material for: Habitual coffee consumption poorly correlates with sleep quality and daytime sleepiness: A cross-sectional study
Source: PLoS One. 2026 Mar 9;21(3):e0344479. doi: 10.1371/journal.pone.0344479 (PMC12970861; doi:10.1371/journal.pone.0344479)
Supplement: S4 Table — Four models were fitted: Model 1 (M1) with coffee consumption as a four-category variable, Model 2 (M2) with coffee consumption as the original questionnaire categories, Model 3 (M3) with coffee consumption modeled as a continuous variable using numeric approximate midpoints for each outcome category under the assumption of a linear association, and Model 4 (M4) with coffee consumption modeled continuously using restricted cubic splines to allow for non-linear associations. M1 and M2 were compared to test whether collapsing the original coffee consumption categories into 4 groups causes meaningful information loss. M3 and M4 were compared to assess whether a model that allows non-linearity fits better to the data. Additional model metrics were also calculated, including Akaike Information Criterion (AIC), Bayesian Information Criterion (BIC), and Nagelkerke pseudo R-squared (R2). For quasi-Poisson models, Quasi-Akaike Information Criterion (QAIC) and deviance are given. (DOCX) [file pone.0344479.s010.docx]

***S4 Table.*** ***Sensitivity analysis results for assessing non-linearity.*** *Four models were fitted: Model 1 (M1) with coffee consumption as a four-category variable, Model 2 (M2) with coffee consumption as the original questionnaire categories, Model 3 (M3) with coffee consumption modeled as a continuous variable using numeric approximate midpoints for each outcome category under the assumption of a linear association, and Model 4 (M4) with coffee consumption modeled continuously using restricted cubic splines to allow for non-linear associations. M1 and M2 were compared to test whether collapsing the original coffee consumption categories into 4 groups causes meaningful information loss. M3 and M4 were compared to assess whether a model that allows non-linearity fits better to the data. Additional model metrics were also calculated, including Akaike Information Criterion (AIC), Bayesian Information Criterion (BIC), and Nagelkerke pseudo R-squared (R2). For quasi-Poisson models, Quasi-Akaike Information Criterion (QAIC) and deviance are given.*

| Outcome | M1 vs M2 | M3 vs M4 | AIC | BIC | R2 | QAIC | Deviance |
| --- | --- | --- | --- | --- | --- | --- | --- |
| SleepTime | 0.006855025 | 6.22E-10 | 70041.44 | 70228.7 | 0.023388 | NA | NA |
| SleepQuality | 1.35E-09 | 0.000701821 | 67370.63 | 67541.6 | 0.13884 | NA | NA |
| DifficultFallAsleep | 1.37E-08 | 0.04393168 | 66164.07 | 66335.05 | 0.089321 | NA | NA |
| WakingUp | 0.001426863 | 0.039612874 | 79258.36 | 79429.34 | 0.076577 | NA | NA |
| WakingUpEarly | 6.18E-07 | 0.039984494 | 72327.91 | 72498.88 | 0.062315 | NA | NA |
| Reflux | 0.050188862 | 0.983974074 | 37376.73 | 37547.71 | 0.064174 | NA | NA |
| Snoring | 0.316518915 | 0.137709733 | 73156.93 | 73327.9 | 0.134665 | NA | NA |
| Sleep score | 3.00E-11 | 3.48E-05 | NA | NA | 0.308212 | 25399 | 49159.69 |
| ESS score | 0.00461412 | 1.23E-06 | NA | NA | 0.100334 | 25400 | 70350.63 |
