# Supplementary material for: Habitual coffee consumption poorly correlates with sleep quality and daytime sleepiness: A cross-sectional study
Source: PLoS One. 2026 Mar 9;21(3):e0344479. doi: 10.1371/journal.pone.0344479 (PMC12970861; doi:10.1371/journal.pone.0344479)
Supplement: S5 Table — Adjusted predicted probabilities for sleep categories based on coffee consumption level and signed absolute differences between coffee consumption groups. For sleep score and ESS score, values are given as predicted counts. (DOCX) [file pone.0344479.s011.docx]

***S5 Table.*** ***Adjusted predicted probabilities.*** *Adjusted predicted probabilities for sleep categories based on coffee consumption level and signed absolute differences between coffee consumption groups. For sleep score and ESS score, values are given as predicted counts.*

|  | **Predicted probabilities** | | | |  | **Signed absolute differences** | | |
| --- | --- | --- | --- | --- | --- | --- | --- | --- |
|  | NONE | LOW | MODERATE | HIGH |  | LOW-NONE | MODERATE-  NONE | HIGH-NONE |
| **SleepTime** | | | | | | | | |
| 5 hours | 0.981 | 0.985 | 0.990 | 0.990 |  | 0.004 | 0.008 | 0.009 |
| 6 hours | 0.921 | 0.920 | 0.942 | 0.937 |  | −0.001 | 0.021 | 0.016 |
| 7 hours | 0.698 | 0.696 | 0.737 | 0.699 |  | −0.001 | 0.039 | 0.001 |
| 8 hours | 0.254 | 0.287 | 0.265 | 0.216 |  | 0.033 | 0.011 | −0.038 |
| 9 hours | 0.060 | 0.075 | 0.046 | 0.031 |  | 0.015 | −0.014 | −0.029 |
| 10 hours or more | 0.009 | 0.020 | 0.009 | 0.005 |  | 0.011 | 0.000 | −0.003 |
| **SleepQuality** | | | | | | | | |
| Good | 0.895 | 0.901 | 0.894 | 0.877 |  | 0.006 | −0.001 | −0.019 |
| Somewhat good | 0.613 | 0.661 | 0.621 | 0.588 |  | 0.048 | 0.008 | −0.025 |
| Bad | 0.225 | 0.243 | 0.194 | 0.181 |  | 0.018 | −0.031 | −0.043 |
| Very bad | 0.036 | 0.043 | 0.025 | 0.022 |  | 0.007 | −0.011 | −0.014 |
| **DifficultFallAsleep** | | | | | | | | |
| Less than once per week | 0.774 | 0.799 | 0.783 | 0.755 |  | 0.025 | 0.010 | −0.019 |
| 1-2 times/week | 0.519 | 0.554 | 0.502 | 0.481 |  | 0.035 | −0.016 | −0.037 |
| 3-6 times/week | 0.264 | 0.292 | 0.226 | 0.225 |  | 0.028 | −0.038 | −0.039 |
| Almost every night | 0.138 | 0.168 | 0.104 | 0.104 |  | 0.030 | −0.035 | −0.035 |
| **WakingUp** | | | | | | | | |
| Less than once per week | 0.823 | 0.865 | 0.853 | 0.834 |  | 0.041 | 0.030 | 0.011 |
| 1-2 times/week | 0.640 | 0.676 | 0.659 | 0.635 |  | 0.036 | 0.019 | −0.005 |
| 3-6 times/week | 0.445 | 0.469 | 0.433 | 0.419 |  | 0.024 | −0.012 | −0.027 |
| Almost every night | 0.285 | 0.306 | 0.255 | 0.245 |  | 0.021 | −0.030 | −0.040 |
| **WakingUpEarly** | | | | | | | | |
| Less than once per week | 0.648 | 0.678 | 0.640 | 0.601 |  | 0.030 | −0.007 | −0.047 |
| 1-2 times/week | 0.358 | 0.374 | 0.335 | 0.310 |  | 0.016 | −0.023 | −0.048 |
| 3-6 times/week | 0.159 | 0.163 | 0.135 | 0.122 |  | 0.003 | −0.025 | −0.038 |
| Almost every night | 0.060 | 0.056 | 0.044 | 0.039 |  | −0.004 | −0.016 | −0.022 |
| **Reflux** | | | | | | | | |
| Less than once per week | 0.214 | 0.237 | 0.206 | 0.184 |  | 0.023 | −0.009 | −0.030 |
| 1-2 times/week | 0.080 | 0.097 | 0.078 | 0.066 |  | 0.017 | −0.002 | −0.014 |
| 3-6 times/week | 0.031 | 0.038 | 0.026 | 0.021 |  | 0.007 | −0.005 | −0.010 |
| Almost every night | 0.010 | 0.010 | 0.009 | 0.007 |  | 0.000 | −0.001 | −0.003 |
| **Snoring** | | | | | | | | |
| Seldom | 0.807 | 0.850 | 0.860 | 0.872 |  | 0.043 | 0.053 | 0.065 |
| Sometimes | 0.525 | 0.549 | 0.562 | 0.572 |  | 0.023 | 0.037 | 0.047 |
| Often | 0.193 | 0.198 | 0.197 | 0.212 |  | 0.005 | 0.003 | 0.019 |
| Very often | 0.064 | 0.072 | 0.066 | 0.067 |  | 0.008 | 0.002 | 0.003 |
| **Sleep and sleepiness scores** | | | | | | | | |
| SleepScore | 8.653 | 9.117 | 8.498 | 8.276 |  | 0.464 | −0.155 | −0.377 |
| ESS | 4.074 | 3.840 | 3.754 | 3.893 |  | −0.234 | −0.320 | −0.181 |
