# Supplementary material for: Habitual coffee consumption poorly correlates with sleep quality and daytime sleepiness: A cross-sectional study
Source: PLoS One. 2026 Mar 9;21(3):e0344479. doi: 10.1371/journal.pone.0344479 (PMC12970861; doi:10.1371/journal.pone.0344479)
Supplement: S1 File — (PDF) [file pone.0344479.s012.pdf]

Supplementary information

For manuscript: Habitual coffee consumption poorly correlates with sleep quality and daytime sleepiness: a cross-sectional study

Additional methods details

SCAPIS

The Swedish Cardiopulmonary Bioimage Study (SCAPIS) is the largest cardiovascular and pulmonary research program in Sweden to date, a multicenter collaboration between six host universities and university hospital centers in Sweden (Umeå, Uppsala, Stockholm, Linköping, Gothenburg and Malmö/Lund) conducted between 2013 and 2018 [1]. It comprises a wide range of clinical variables as well as genetic data, characterizing a Swedish cohort of 30,154 randomly selected individuals aged 50 to 64.

Genetics

The large-scale genotyping effort of SCAPIS has been described in detail elsewhere. Briefly, whole blood DNA samples were extracted from the Karolinska Institute biobank (https://ki.se/en/research/ki-biobank) and genotyped at the SNP&SEQ Technology Platform in Uppsala (www.genotyping.se). Genotyping was done in 10 batches using the Illumina GSA-MDv3 (customized version) and genotypes were called with GenomeStudio 2.0.3. A total of 726,358 genetical markers (single-nucleotide polymorphisms, SNPs) were genotyped. Quality control was performed for sex inconsistencies, call rates and heterozygosity, and principal component analysis was performed to account for population structure and batch effects. Samples were then imputed to the Haplotype Reference Consortium (HRC) r1.1 reference panel [2] at the Sanger imputation service. The resulting imputed genotype dataset comprises 29,335 samples and ~40 million SNPs (reference genome build 37 positions).

GWAS

The imputation procedure, described in the SCAPIS section above, reports an information metric (info score) that represents the confidence of imputed SNPs. This metric typically takes values between 0 and 1, where values near 1 indicate that a SNP has been imputed with high certainty. SNPs with an info score > 0.3 and a mean allele frequency (MAF) > 1% were selected, resulting in a total of ~8 million SNPs. The data, initially in chromosome-separated files, was also merged into a single .pgen file set before further analysis.

Data merging, filtering and GWAS were performed using PLINK 2.0 [3], an open-source whole genome association analysis toolset (www.cog-genomics.org/plink/2.0/), with the following settings: "--glm hide-covar firth-fallback firth-residualize single-prec-cc --covar-variance-standardize". Visualization of GWAS results as Manhattan plots and genomic region plots were made using the R package topr 2.0.0 [4].

References

[1] G. Bergström *et al.*, 'The Swedish CArdioPulmonary BioImage Study: Objectives and design', *J. Intern. Med.*, vol. 278, no. 6, pp. 645–659, Dec. 2015, doi: 10.1111/joim.12384.

[2] S. McCarthy *et al.*, 'A reference panel of 64,976 haplotypes for genotype imputation', *Nat. Genet.*, vol. 48, no. 10, pp. 1279–1283, Oct. 2016, doi: 10.1038/ng.3643.

[3] C. C. Chang, C. C. Chow, L. C. A. M. Tellier, S. Vattikuti, S. M. Purcell, and J. J. Lee, 'Second-generation PLINK: Rising to the challenge of larger and richer datasets', *Gigascience*, vol. 4, no. 1, Feb. 2015, doi: 10.1186/s13742-015-0047-8.

[4] T. Juliusdottir, 'topr: an R package for viewing and annotating genetic association results', *BMC Bioinformatics*, vol. 24, no. 1, Dec. 2023, doi: 10.1186/s12859-023-05301-4.
