# Supplementary material for: Habitual coffee consumption poorly correlates with sleep quality and daytime sleepiness: A cross-sectional study
Source: PLoS One. 2026 Mar 9;21(3):e0344479. doi: 10.1371/journal.pone.0344479 (PMC12970861; doi:10.1371/journal.pone.0344479)
Supplement: Attachment — Submitted filename: 20250814_Response_to_Comments_PONE.docx [file pone.0344479.s013.docx]

Dear editor at PLOS ONE,

We are pleased to submit our manuscript, entitled “Habitual coffee consumption poorly correlates with sleep quality and daytime sleepiness: a cross-sectional study” for your consideration. In a previous submission (PONE-D-25-10508), significant limitations were pointed out. These comments have been of great value to us in refining our work, and we hereby present a considerably strengthened manuscript that addresses all of them. For your convenience, we report the comments received in the previous submission followed by an account of how we have addressed them.

We are looking forward to hearing from you.

Best wishes,

Simon Söderholm

DETAILED RESPONSE TO COMMENTS

Below we explain in detail how we have addressed the concerns raised (editor’s comments are displayed in red, and our responses in black).

-----

After internal review of your manuscript, we had several concerns with the analyses, conclusions and quality of the scientific reporting. Please note that as per our publication criteria, PLOS ONE requires that all experiments, statistics and other analyses are performed to a high technical standard, described in sufficient detail and adhere to appropriate reporting guidelines and community standards. Conclusions must be presented in an appropriate fashion and be supported by the data (Please see http://journals.plos.org/plosone/s/criteria-for-publication). After careful consideration, we feel that the current manuscript does not meet these requirements..

We are grateful for the careful assessment of our work. The feedback received has allowed us to revise our study and address the criticism raised. Specifically, we have revisited and refined our analyses, and improved the manuscript by ensuring better reporting of the results in tables and figures.

Specifically, we require mendelian randomization analyses to adhere to rigorous statistical analysis and significance thresholds. This includes, but is not limited to the inclusion of sample size/power calculation, appropriate statistical tests and strongly independent genetic instruments, that are robustly associated with the risk factor of interest. While we understand that these thresholds can vary, we would expect the methods section to contain detailed information regarding the choice of p value cut-offs. Additionally, considering the essential requirements of strong IV assumptions, we would expect an assessment if selected genetic variants are associated with potential confounders. While the risk for horizontal or vertical pleiotropy can be estimated using adequate statistical tests, we also require a thorough evaluation of the suitability of these SNPs based on published literature. We are concerned that your methodology does not fulfill those criteria, and as such we have concerns about the ability of the study design employed to support the conclusions made.

We fully agree with this point. While the mendelian randomization analysis we employed was carefully done and entailed the necessary steps to ensure that informative SNPs were selected, it lacked some important components as pointed out by the editor. Therefore, and as the contribution of this analysis towards the study’s overall aim was limited, we decided to remove it. Instead, we have put more emphasis on our other analyses. This includes a refinement of our regression analysis, where the regression methods have been adjusted to better suit the data at hand. Specifically, we analyzed the relationship between coffee consumption and each separate sleep factor using ordinal regression modelling, while quasi-Poisson generalized linear models were used when assessing the relationship between coffee consumption and ESS/Sleep score.

We have also added an additional analysis in which we divided the study population, based on their genotype of the caffeine metabolism related SNP rs762551, into “slow” and “fast” metabolizers, similar to what have previously been presented in a study by Zhou and Hyppönen (2019) (PMID:30838377). We then assessed the relation between coffee consumption and sleep/ESS through regression analysis on each of these two metabolism-groups.

Lastly, we have improved the overall reporting. Figure 1 now gives a more informative overview of the study, including an updated flow chart of the main analysis steps. Also, results are now more clearly presented in structured tables. Specifically, Table 1 gives an overview of the coffee consumption data and baseline characteristics early in the results section. Taken together, these changes have, in our opinion, resulted in an improved and more streamlined manuscript.
