# Supplementary material for: Habitual coffee consumption poorly correlates with sleep quality and daytime sleepiness: A cross-sectional study
Source: PLoS One. 2026 Mar 9;21(3):e0344479. doi: 10.1371/journal.pone.0344479 (PMC12970861; doi:10.1371/journal.pone.0344479)
Supplement: Attachment — Submitted filename: 20260130_Response_to_Reviewers_PONE.docx [file pone.0344479.s014.docx]

Dear Dr. da Costa,

We are pleased to submit our revised manuscript, entitled “Habitual coffee consumption poorly correlates with sleep quality and daytime sleepiness: a cross-sectional study” for your consideration. We are grateful for the thorough review of our work, thanks to which we now present a considerably strengthened manuscript. Specifically, we have:

- Refined our analyses by including previously overlooked diagnostics tests and sensitivity analyses, and added absolute risk differences for easier interpretation.
- Improved our statistical models by adding sleep medication as covariate and adjusting the sleep score calculation.
- Revised main statements and conclusions to better reflect the limitations of the study.

For your convenience, we report the reviewers’ remarks followed by a detailed account of how we have addressed each of them.

We are confident that implementing our response to the criticisms and suggestions received has improved the clarity of our article and the strength of our findings. Thank you for offering us the opportunity to resubmit our manuscript. We are looking forward to hearing from you.

Best wishes,

Simon Söderholm

DETAILED RESPONSE TO THE REVIEWERS

We are grateful to the referees for their detailed evaluation of our work, the data and the resulting conclusions. By integrating their comments and suggestions we were able to improve our analyses and strengthen our findings. Below we explain in detail how we have addressed each point raised (reviewers’ comments are displayed in red, and our responses in black).

-----

Academic Editor:

1. Please ensure that your manuscript meets PLOS ONE's style requirements, including those for file naming.

Thank you for pointing out the style requirements of the journal. We have now gone through our manuscript and made the necessary changes to comply with these requirements. The figures have been removed from the main manuscript and uploaded as individual TFF files. Figure captions have been added in the manuscript text directly after their first mentioning. Supplementary figures are still found in the supplementary material file.

2. Please state what role the funders took in the study.  If the funders had no role, please state: "The funders had no role in study design, data collection and analysis, decision to publish, or preparation of the manuscript." If this statement is not correct you must amend it as needed. Please include this amended Role of Funder statement in your cover letter; we will change the online submission form on your behalf.

The funders have not been involved in any part of the study or manuscript. Hence, the statement “The funders had no role in study design, data collection and analysis, decision to publish, or preparation of the manuscript.” has been added to the funding section of the manuscript.

3. We note that you have indicated that there are restrictions to data sharing for this study. For studies involving human research participant data or other sensitive data, we encourage authors to share de-identified or anonymized data. However, when data cannot be publicly shared for ethical reasons, we allow authors to make their data sets available upon request. If there are ethical or legal restrictions on sharing a de-identified data set, please explain them in detail (e.g., data contain potentially identifying or sensitive patient information, data are owned by a third-party organization, etc.) and who has imposed them (e.g., a Research Ethics Committee or Institutional Review Board, etc.). Please also provide contact information for a data access committee, ethics committee, or other institutional body to which data requests may be sent. If there are no restrictions, please upload the minimal anonymized data set necessary to replicate your study findings to a stable, public repository and provide us with the relevant URLs, DOIs, or accession numbers. Please see http://www.bmj.com/content/340/bmj.c181.long for guidelines on how to de-identify and prepare clinical data for publication. For a list of recommended repositories, please see https://journals.plos.org/plosone/s/recommended-repositories. You also have the option of uploading the data as Supporting Information files, but we would recommend depositing data directly to a data repository if possible. Please update your Data Availability statement in the submission form accordingly.

The data underlying the current study derives from the Swedish CArdioPulmonary bioImage Study (SCAPIS), a multi-center study conducted at six university hospitals in Sweden, examining 30,154 randomly selected men and women aged 50-64 years. The first public release of data was on March 17, 2021, available to be applied for by researchers based in Sweden or international researchers in collaboration with a researcher based in Sweden. Applicants need to be connected to a research institution in Sweden and need an approval from the Swedish Ethical Review Board (Etikprövningsmyndigheten). Furthermore, regarding international data sharing, SCAPIS data is subject to confidentiality under Chapter 24, Section 8 of the Swedish Public Access to Information and Secrecy Act (OSL), and Section 7 of the Public Access to Information and Secrecy Ordinance.

Based on the information above, we are unfortunately unable to publicaly share any raw data at this point. Processed data have been made available in tables both in the main manuscript as well as in supplementary materials. We have now extended the data availability statement in the manuscript with the above information.

For more information regarding access to SCAPIS data please visit the SCAPIS homepage at <https://www.scapis.org/data-access/>

4. Your ethics statement should only appear in the Methods section of your manuscript. If your ethics statement is written in any section besides the Methods, please move it to the Methods section and delete it from any other section. Please ensure that your ethics statement is included in your manuscript, as the ethics statement entered into the online submission form will not be published alongside your manuscript.

The ethics statement has now been moved to the methods section of the manuscript.

5. If the reviewer comments include a recommendation to cite specific previously published works, please review and evaluate these publications to determine whether they are relevant and should be cited. There is no requirement to cite these works unless the editor has indicated otherwise.

The reviewers have not given any recommendations regarding specific publications.

Reviewer #1:

Thank you for the opportunity to review this manuscript. The authors investigate the association between habitual coffee consumption and sleep quality/daytime sleepiness in a large Swedish cohort (SCAPIS, n≈25,000), complemented by genetic validation of self-reported coffee intake. The topic is timely and relevant, given the widespread consumption of caffeine and its disputed long-term effects on sleep. The sample size and the genetic analysis are strengths. However, several methodological and reporting issues limit the interpretability and impact of the study. Below I provide my detailed comments.

We are grateful for the interest shown in our work and findings. We have carefully considered the criticisms proposed. These made us implement changes that now render our analyses - and the study overall, we strongly believe - considerably stronger.

Critique

Mayor concerns:

1. Study design and causal inference

The cross-sectional design is suitable for descriptive associations but inherently limited for causal inference. The discussion should more explicitly acknowledge that the analyses cannot establish long-term effects of coffee on sleep or sleepiness.

We fully agree that the cross-sectional design of our study does not permit complete causal inference to be made. We have now addressed this more clearly in the beginning of the study limitations section of the manuscript. We also want to mention that a continuation of the SCAPIS study, named SCAPIS2, in which half of the original participants (15.000) undergo another round of comprehensive medical examinations, has recently been initiated. Thus, we consider our current study results a valuable foundation for a follow-up longitudinal study which we aim to conduct using the data from SCAPIS2.

2. Handling of missing data

The authors excluded all participants with any missing data (complete-case analysis). This approach may introduce selection bias if data are not missing completely at random. A description of the missingness patterns and, ideally, multiple imputation or sensitivity analyses are required.

We did conduct some initial tests with imputation of missing data, but the reliability of this proved to be inadequate, particularly regarding coffee consumption. However, it is true that we never conducted any further analyses of the nature of the missing data. To address this, we have now conducted a more in-depth investigation of our missing data and the possibility of imputation.

Considering all the 25 variables included in our study (coffee consumption, sleep factors, the eight ESS-related criteria, and identified confounders), the proportion of missing data values is approximately 3.4% across the entire dataset. Little’s MCAR test indicated that the data is not missing completely at random (MCAR), but either missing at random (MAR) or missing not at random (MNAR) (p < 0.001), suggesting that there may be patterns in data missingness. This is, however, expected, as participants who have not answered a question on sleep habits more likely have not answered the other questions on sleep either. Indeed, looking at the correlation of missing data between all variables, sleep factors are correlated with each other, as is the case for ESS criteria. In contrast, coffee consumption, while showing 100% overlap of missing values with that of tea consumption, does not correlate with missing data in any of the other variables.

This indicates that, while removing participants with missing data in any of the sleep or ESS-related variables could potentially introduce selection bias, this would not be the case for coffee consumption.

Next, we set out to again investigate whether the missing data could reliably be imputed. Treating all variables as categorical (with age and BMI divided into categorical groups) we performed multiple imputation by multiple correspondence analysis (MCA). To evaluate imputation quality, we computed the proportion of correctly imputed values (accuracy) for each variable. To contextualize this performance, we compared it to the expected accuracy under random guessing based on marginal category frequencies (“naive baseline”), and expressed this as a ratio. This ratio can be interpreted as a relative improvement measure, similar to those used in simulation-based imputation evaluations (Josse & Husson, 2016; Audigier et al., 2016).

In the figure below, imputation accuracy is plotted for each variable in the form of proportion of correctly imputed value (left panel) and the ratio between this proportion and the expected accuracy under random guessing (right panel). For many of the variables, including coffee consumption, the ratio is close to 1, meaning that the imputation did not perform better than random guessing. While slightly higher ratios were observed for some of the sleep and ESS variables, they still correspond to just a small improvement compared to random guessing.

In conclusion, these results, in our opinion, suggest that the data at hand may not be suitable for imputation, at least not according to the imputation method deployed here. While there are other methods available, multiple imputation by MCA constitute, to our knowledge, a sophisticated procedure (compared to more simple techniques like mean/mode imputation and listwise deletion), and is also well applicable to categorical data. In addition, while correlation in missing data was observed between sleep factors and ESS criteria, this is expected, as these derive from closely related questions in the questionnaire, and therefore, we believe, may not confer any relevant selection bias upon deletion. Missing data on coffee consumption showed complete correlation with that of tea consumption (i.e. participants that did not answer the coffee question also never answered the tea question). This, together with the fact that missing data in coffee consumption did not correlate with that of any other variable, indicates that imputation of missing data on coffee consumption is neither easy to do nor necessary for the subsequent analyses.

The results from this missingness analysis are presented in supplementary materials, Figure S1. The method section of the manuscript has also been amended accordingly.

3. Exposure measurement (coffee consumption)

Coffee intake was captured through a food frequency questionnaire and grouped into four categories. This grouping collapses possible non-linear associations (e.g., U-shaped curves) and may obscure dose–response effects.

The main reason for our aggregation of the original coffee consumption groups regards the sparsity of the data. The number of participants varies greatly across the original coffee consumption groups, with some groups containing very few participants (Figure 1C). This can render model estimates (especially in generalized linear models) unstable, with wide confidence interval or convergence problems, and can distort effect estimates and inflate standard errors. Collapsing categories reduce noise and prevent model overfitting.

However, we understand the concern raised by the reviewer. While group aggregation is, in our opinion, justified given the unbalanced data, it may obscure non-linear dose-dependent patterns. To address this, we have now employed a sensitivity analysis. For each outcome (i.e. sleep factor, sleep score and ESS), we fitted four different models:

| Model | Description |
| --- | --- |
| M1 | With coffee consumption aggregated into four groups as in the main analysis |
| M2 | With the original 10 coffee consumption groups |
| M3 | With coffee consumption as a continuous variable |
| M4 | With coffee consumption as a continuous variable, using restricted cubic splines for flexible modelling, which can capture non-linear associations |

Coffee consumption as a continuous variable was defined as the approximate midpoint of each category (times/day) according to below:

| Original coffee category | Approx. midpoint (times/day) |
| --- | --- |
| No consumption | 0 |
| 1–3 times/month | 0.07 |
| 1–2 times/week | 0.21 |
| 3–4 times/week | 0.5 |
| 5–6 times/week | 0.8 |
| 1 time/day | 1 |
| 2 times/day | 2 |
| 3 times/day | 3 |
| 4 times/day | 4 |
| 5 times/day or more | 5 |

As before, cumulative link models were used for ordinal sleep variables and quasi-Poisson models were used for ESS and the sleep score. For each of the four models (M1, M2, M3 and M4) and for each of the outcomes, predicted probabilities were computed and visualized.

Next, for each outcome, models were compared to assess improvememnt in model fit, using likelihood ratio tests for ordinal models and F-tests for the quasi-Poisson models. M1 and M2 were compared to test whether collapsing the original coffee consumption categories into 4 groups causes meaningful information loss. M3 and M4 were compared to assess whether a model that allows non-linearity fits better to the data. Some additional model metrics were also calculated, including Akaike Information Criterion (AIC), Bayesian Information Criterion (BIC), and Nagelkerke pseudo R-squared (R^2^). For quasi-Poisson models, Quasi-Akaike Information Criterion (QAIC) and deviance are given. The results are presented in a summary table below:

| outcome | M1 vs M2 | M3 vs M4 | AIC | BIC | R2 | QAIC | Deviance |
| --- | --- | --- | --- | --- | --- | --- | --- |
| SleepTime | 0.006855025 | 6.22E-10 | 70041.44 | 70228.7 | 0.023388 | NA | NA |
| SleepQuality | 1.35E-09 | 0.000701821 | 67370.63 | 67541.6 | 0.13884 | NA | NA |
| DifficultFallAsleep | 1.37E-08 | 0.04393168 | 66164.07 | 66335.05 | 0.089321 | NA | NA |
| WakingUp | 0.001426863 | 0.039612874 | 79258.36 | 79429.34 | 0.076577 | NA | NA |
| WakingUpEarly | 6.18E-07 | 0.039984494 | 72327.91 | 72498.88 | 0.062315 | NA | NA |
| Reflux | 0.050188862 | 0.983974074 | 37376.73 | 37547.71 | 0.064174 | NA | NA |
| Snoring | 0.316518915 | 0.137709733 | 73156.93 | 73327.9 | 0.134665 | NA | NA |
| Sleep score | 3.00E-11 | 3.48E-05 | NA | NA | 0.308212 | 25399 | 49159.69 |
| ESS score | 0.00461412 | 1.23E-06 | NA | NA | 0.100334 | 25400 | 70350.63 |

While the results imply loss of information from collapsing coffee categories (M1 vs M2 p < 0.05) and the existence of a possible U-shaped association (M3 vs M4 p < 0.05), visual inspection of predicted probabilities (below) suggests non-linear patterns to be mild, and that collapsing coffee consumption groups still capture these patterns. Statistical significance is probably due to the large sample size.

Overall, we assess that our initial analysis using aggregated coffee consumption categories does not overlook possible non-linear patterns and associations. We therefore consider our results robust.

The results from this analysis are now presented as Figure S4 Table S4 in supplementary materials. Note that due to implementation of other changes to the overall analysis workflow (use of sleep medication as a covariate, and inclusion of sleep duration in the sleep score calculation) the numbers of the non-linearity analysis in Figure S4 differ a bit from those shown here. However, these are just minor differences that do not change our assessment.

The “high” group is labeled as 4–5 cups/day, but it seems to include “≥5/day” as well; please clarify.

Thank you for the thorough review of our text. This is indeed an ambiguity on our part. The “high” coffee consumption group consists of 4-5 times/day or more, which we now have better stated in the method section.

No information is available on decaffeinated coffee, timing of consumption (morning vs. evening), or other caffeine sources (energy drinks, sodas). These are important determinants of caffeine’s effect on sleep and should at least be discussed.

The reviewer is right in pointing out the lack of certain information on the consumption of coffee. Regarding the consumption of decaffeinated coffee, it would definitely have been a useful factor to include in our analysis. Decaffeinated coffee has seen a rise in popularity in recent years, especially among younger individuals. Unfortunately, decaffeinated coffee was not included as a response option in the SCAPIS questionnaire. This was also true for energy drinks and sodas, which we acknowledge in our study limitation statement in the discussion section of the manuscript in the following way: “In addition, it is possible that participants may consume caffeine from sources other than coffee or tea, such as energy drinks or certain soft drinks. Data on the consumption of such drinks was not included in this study. However, although we cannot rule out some effect of these neglected caffeine sources, it is likely that coffee and tea are the main sources, as their consumption tends to have a more consistent and habitual pattern."

The timing of coffee intake is also a factor of importance, especially regarding the potential effect on nighttime sleep. However, the magnitude of this effect is, in our opinion, debatable, considering that caffeine is reported to reach its peak concentration in blood within about 30 to 60 minutes after consumption and that its mean half-life is 3 to 5 hours. Some factors are of course known to influence the half-life of caffeine. This included smoking, which we have adjusted for in our analysis. Nonetheless, it is possible that participants consume coffee close to bedtime, which may constitute a confounding factor not accounted for in our analysis. We have therefore stated this limitation more clearly in the discussion section of the manuscript.

4. Sleep variables: description and use

Seven items were collected (hours of sleep, sleep quality, sleep latency, nocturnal awakenings, early awakening, reflux, loud snoring). Most were rated on 0–4 Likert scales, but “hours of sleep” was recorded on a different scale.

The authors constructed a composite “sleep score” summing six items and *excluded hours of sleep*. They justify this on grounds of different scaling and considering duration as preference rather than quality. While defensible, sleep duration is a central dimension of sleep health and strongly linked to sleepiness. Excluding it weakens the construct validity of the score.

For robustness, I strongly recommend sensitivity analyses that incorporate hours of sleep (after standardization), or alternatively provide two scores: “sleep quality” (without duration) and “sleep health” (with duration).

Yes, initially we decided not to include sleep duration as a component of the sleep score due to its different scale and our assumption that hour of sleep may be influenced by individual preference in addition to overall sleep quality. However, the reviewer is right that sleep duration is an important aspect of sleep health. It is also closely linked to daytime sleepiness, which was the main reason we included adjustment for sleep duration when we modelled the relationship between ESS and coffee consumption. Therefore, we have now incorporated sleep duration in our sleep score. 7 or more hours of sleep was given a score of 0, 6 hours of sleep was given a score of 1, 5 hours of sleep was given a score of 2, and 4 hours or less was given a score of 3. These were then added the previous sleep score for each participant. These new sleep scores, now with sleep duration included, were then used in all subsequent analyses.

Furthermore, the methods should describe how hours of sleep were categorized or treated in models. This is insufficiently specified.

Hours of sleep were, as other sleep factors, self-reported through a questionnaire, and categorized into seven groups: “4 hours or less”, ”5 hours”, ”6 hours”, ”7 hours”, ”8 hours”, ”9 hours”, and ”10 hours or more”. As for the other sleep factors, it was treated as an ordinal categorical outcome when a cumulative link model was fitted. In addition, when modelling ESS as outcome, hours of sleep were included as one of the confounders adjusted for in the model. Hours of sleep were also recategorized for inclusion in the sleep score calculation (see response to previous comment). In our revised supplementary materials, we have now added a table (Table S1) with details on each of the variables included in the study, their original collection and use.

5. Statistical modeling

Use of cumulative link models for ordinal sleep variables and quasi-Poisson regression for ESS and the composite score is appropriate. However, the manuscript does not report diagnostic checks, particularly the proportional odds assumption in ordinal models. This is a major omission.

We agree that appropriate diagnostic tests might have been overlooked in the manuscript. Accordingly, we have now added such analyses. The proportional odds (PO) assumption was assessed using the nominal test and scale test from the ordinal R package. The figure below shows the statistical significance obtained by these two tests for each of the modeled sleep traits (y-axis) and each predictor/covariate included in the models (x-axis).

As evident from the figure, these tests were significant for several of the variables, which suggest violation of the PO assumption. However, due to our large sample size (N > 25,000), this can cause these statistical tests to become overly sensitive, detecting small and inconsequential deviations from proportional odds. In addition, health-related ordinal variables tend to have uneven spacing, where different behavior of an outcome’s levels based on a predictor could be expected. To investigate whether the violation is substantive, we fitted partial proportional odds models, using the same formulas and covariates, and computed threshold-specific odds ratios and cumulative logit. While these two metrics also pointed to a violation of the PO assumption, this only translates to small differences in predicted category probabilities (min: 0.0002, 1^st^ quantile: 0.0086, median: 0.0187, mean: 0.0233, 3^rd^ quantile: 0.0359, max: 0.0766). Therefore, although statistical non-proportionality was detected, it is most likely not of practical relevance. Thus, we decided to keep our proportional-odds model. The results from the above PO assumption analysis are presented in supplementary materials, figure S3, including statistical tests (panel A), cumulative logit (panel B), threshold-specific odds ratios (panel C), and predicted probabilities (panel D). The methods and results sections of the manuscript have been amended accordingly.

For goodness of fit checks, please refer to our response to previous comments on non-linearity.

ESS is bounded (0–24) and often skewed. Modeling it as quasi-Poisson is unusual; linear robust regression or negative binomial could be tested in sensitivity analyses. Categorizing ESS (≤10, 11–12, ≥13) might also aid interpretability.

As we found the ESS data to exhibit overdispersion, due to its skewness, we decided to fit a quasi-Poisson model rather than a standard Poission model. While we would argue that the ESS score distribution does not fully represent a negative binomial distribution, it is possible that the skewness towards lower values could indeed approximate such a distribution. Therefore, we agree that a sensitivity analysis is advisable. To this end, we fitten a negative binomial model and a robust linear regression model, using the same formula and covariates as previously with ESS score as outcome and coffee consumption groups as main predictor. Predicted ESS counts across coffee consumption groups were essentially indistinguishable between the quasi-Poisson and the negative binomial models (see figure below). While predicted ESS counts for the robust linear model did differ slightly in magnitude, the standard error was higher and the overall trend followed the same pattern as for the other models. Based on these results, we decided to keep the quasi-Poisson model for ESS.

Multiple comparisons: with several sleep factors and models tested, there is a risk of type I error inflation. The authors should clarify whether they applied any correction or provide justification for not doing so.

The multiple comparison problem and type I error inflation is indeed an important consideration in many biostatistical approaches. However, in our study we tested a total of nine models (one for each of the seven sleep factors, one for sleep score, and one for ESS), which we consider to be well below the number at which the multiple comparison problem becomes an issue at a significance level of p < 0.05.

6. Clinical relevance versus statistical significance

With n>25,000, even trivial associations reached p<0.05 (e.g., Kendall’s tau = –0.045). The manuscript must emphasize that effect sizes are extremely small and not clinically meaningful. Reporting standardized effect sizes and absolute risk differences would improve clarity.

Yes, we agree on this point. Given our large sample size, statistical significance can indeed be reached even when the association is very weak. As pointed out, this is evident both from the correlation and the regression analyses. Based on these results, we concluded that the level of habitual coffee consumption, as self-reported by the participants, shows low or no association with estimates of sleep quality or daytime sleepiness in the studied population.

However, we also agree that our results reporting lacks some clarity, and we have therefore now improved our analysis. Specifically, while we choose to retain our proportional-odds models as main analysis, we decided to use partial proportional odds models (which do not assume proportional odds) and calculate absolute risk differences for better interpretation. Across the seven sleep variables we studied, absolute differences in predicted category probabilities between coffee consumption groups were small overall (median 1.9%, IQR 0.9-3.6%, maximum 7.7%). For Sleep score and ESS the differences in predicted counts between coffee consumption groups were also very small (minimum -0.84, median -0.2, IQR -0.33-(-0.05), maximum 0.46), in line with our previous assessment. Results from main coffee consumption comparisons (i.e. High vs. None, Moderate vs. None, and Low vs. None), in the form of signed predicted probability/count differences, are presented in an updated Figure 3, and the full results data are included as supplementary Table S5. The results, methods and discussion sections of the manuscript have been amended accordingly.

7. Confounding and covariates

The DAG approach to select confounders is commendable. However, important potential confounders were omitted, notably medical comorbidities and use of sleep medications. These could substantially bias associations and should be at least acknowledged more fully.

Yes, we certainly agree that these two aspects could be important covariates, which we have overlooked in our analysis. We examined our dataset and identified participants who have reported the use of any of the following sleep medications: melatonin, mirtazapine, propavan, zolpidem, circadin, imovane and zopiklon. In total, 357/25381 (~1.4%) of participants had reported use of any of these medications. While this constitutes a small proportion of our dataset, we decided to include it as a covariate adjusted for in our main regression analysis. Although this entailed some minor changes in obtained metrics and effect sizes, it did not change our initial assessments and conclusions. The method section and limitation discussion has been updated accordingly.

Regarding medical comorbidities, we unfortunately do not have this information readily available for analysis.

Interaction analyses (e.g., by sex, age, BMI) would be informative.

Our study has been focusing on the overall average association between coffee consumption and sleep/sleepiness across all participants. Not including details about subgroups has been a deliberate choice on our part to minimize the risk of introducing too many parallel tracks and thus confusion. However, we find the question interesting and have therefore explored potential effect modification by sex, age and BMI by including interaction terms between coffee consumption and each of these variables in the adjusted models. We then assessed whether the inclusion of these interaction terms improved model fit compared to the original models, by using likelihood ratio tests for ordinal models and F-tests for the quasi-Poisson models. The p-values for each comparison are presented below. Significantly improved model fit (p < 0.05) is highlighted in blue. Note that this analysis was performed as part of the revised workflow in which additional changes has been done according to other reviewer comments (new sleep score calculated with sleep duration included, and use of sleep medication as an added covariate in the models).

According to these results, significantly improved model fit is observed for interaction between coffee consumption and age in relation to ESS score (p = 0.033), interaction between coffee consumption and sex in relation to frequency of waking up (p = 0.045), and interaction between coffee consumption and BMI in relation to difficulty falling asleep (p = 0.002). To investigate whether these significant interactions correspond to differences in effects, model-based estimates were calculated (adjusted predicted probabilities for ordinal models and adjusted predicted counts for the quasi-Poisson models). The only clear effect was that at low coffee consumption there was an increased probability of difficulty falling asleep with increased BMI. These results are presented in supplementary Figure S6.

8. Genetic analysis

The GWAS replicated known loci (AHR, CYP1A1/2), which validates self-reported coffee intake. The stratification by rs762551 (fast vs. slow metabolizers) is interesting but effect sizes were marginal.

The effect sizes for the rs762551 stratification analysis were indeed very small, which is why we concluded that the statistically significant differences most probably are of limited medical impact. However, in our revised manuscript we decided to remove this analysis, as we felt that it did not really fit into the study as a whole and did not contribute much either.

The earlier Mendelian randomization approach has been removed, which is reasonable given prior concerns, but this reduces causal inference. The discussion should acknowledge this explicitly.

Yes, the mendelian randomization analysis was removed because it had certain shortcomings and provided limited contribution towards the study’s overall aim. This reduced causal inference, but this should not be seen as an issue. The principal aim of the study was to investigate the association between coffee consumption and sleep/sleepiness, while establishing causal inference is beyond the scope of the study. We have now clarified this in discussion, specifically in the study limitations section.

9. Data transparency and reproducibility

Data access requires approvals from SCAPIS, which is understandable, but limits reproducibility. The authors should at least ensure that full analysis code and derived data tables are made openly available.

We are sorry for the lack of shareable source data and the impact this has on the reproducibility. The SCAPIS data is available to be applied for by researchers based in Sweden or international researchers in collaboration with a researcher based in Sweden. Applicants need to be connected to a research institution in Sweden and need an approval from the Swedish Ethical Review Board (Etikprövningsmyndigheten). Furthermore, regarding international data sharing, SCAPIS data is subject to confidentiality under Chapter 24, Section 8 of the Swedish Public Access to Information and Secrecy Act (OSL), and Section 7 of the Public Access to Information and Secrecy Ordinance.

Processed data have been made available in tables both in the main manuscript as well as in supplementary materials. In addition, we have now made sure that all the analysis code is available via GitHub (https://github.com/SimonSoderholm/CoffeeSleep-Analysis) and archived on Zenodo (DOI: 10.5281/zenodo.18400906).

minor concerns:

1. Tables are dense and difficult to interpret; simplifying presentation or providing supplementary stratified tables would help.

It is true that some of the tables contain quite a lot of information. We have made every effort to keep layout details as clean as possible and only include relevant data. We do not fully agree with the reviewer that the tables are overly crowded. As part of the revision process, we have added additional supplementary tables, for which we have now tried to make sure that there is sufficient space between rows and columns to make interpretation easier.

2. The discussion repeats background literature without sufficient integration into the present findings. More emphasis on implications for clinical practice and public health is encouraged.

Thank you for pointing this out. We have now reviewed our text and removed unnecessary repetitions, while also adding discussion regarding results from newly added analyses, thereby presenting an improved and more streamlined discussion section.

3. Figures could be streamlined; currently some are overly complex for the message conveyed.

We agree that some of the figures could benefit from some re-working. This may be especially true for Figure 3, where the presentation of predicted probabilities for the fitted models (panel C) is not optimal. We have therefore refined these visualizations to make them more easily interpreted.

4. The manuscript would benefit from clearer differentiation between “sleep quality” and “sleep health,” to avoid confusion for readers.

Regarding our definition of these two aspects of sleep, “sleep quality”, which is the term used in our manuscript, refers to the estimated overall sleep quality as given by the subjective results obtained by the SCAPIS questionnaire. “Sleep health” would, in our opinion, refer to the health-related consequences of sleep patterns. In our manuscript, we only refer to “sleep quality” as our work is focused on the subjective estimates of sleep quality reported by the participants.

The manuscript addresses a relevant question with a strong dataset, but the methodology and interpretation require substantial improvement before being suitable for publication in a high-impact journal.

We are grateful for the detailed review of our work. The comments and suggestions have been very helpful in refining our analyses and the manuscript.

Reviewer #2:

Given the significant limitations in the study (and the limitations in interpretation of the title terms "sleep quality and daytime sleepiness"), the title might be amended to include novel findings in the caffeine-associated GWAS that might stimulate further research.

GWAS was used to test the validity of the self-reported coffee consumption data, and it succeeded in identifying SNPs previously well-known for their association with coffee consumption. The additional associated SNPs not previously reported (of which several were in the genes *CABIN1* and *SUSD2*), while an interesting and potentially novel finding, showed lower overall significance and falls outside of the main scope of the study. We therefore decided to acknowledge it in the discussion, but otherwise leave it a potential direction for future studies.

Major limitation to use of term "sleepiness" in this paper. Sleepiness is the tendency to fall asleep and is measured as mean sleep latency. The correlation between the Epworth Sleepiness Scale and mean sleep latency is relatively poor with r=0.37 (Chervin et al. 1997). Use of this scale as a quantitative measure of sleepiness is problematic. Better to use the term, "subjective estimate of sleepiness."

We agree that the Epworth Sleepiness Scale (ESS), while an established and widely used method for assessing daytime sleepiness (Kendzerska et al. 2014), is based on a self-report questionnaire and therefore does not represent a fully quantitative measure of sleepiness but rather an estimate. To reflect this, we have now made adjustments to our manuscript text in accordance with the suggestion of the reviewer, clarifying that we refer to estimated daytime sleepiness.

The sleep quality score has not been validated against objective measurement of sleep quality, as implied. Reference 41, for validation of the "sleep quality score," derived from the Basic Nordic Sleep Questionnaire, simply refers to reference 40 for validation and can be omitted. It should be noted that in reference 40, the validation of the "sleep quality score" is for cardiovascular disease and it has not been validated with polysomnography as a measure of quality of sleep.

We appreciate the detailed and thorough review of our text, and we agree with the above assessment. The error lies entirely with us, as we failed to ensure the accuracy of the references cited here. Consequently, we have now removed reference 41 and reworded the sentence as follows: “A similar sleep health score has been used in a previous study, although in the context of cardiovascular disease [40]”.

Accuracy of a subject's perceptions of sleep onset and duration can vary widely with actual measurement. As the authors note, caffeine has an effect on cognitive function and therefore, not surprisingly, caffeine consumption distorts one's perception of sleep and sleepiness (e.g., Schlichtiger et al., 2025). Effects of caffeine seem to further confound the already loose relationships among caffeine consumption, subjective estimates of sleepiness and sleep quality (questionnaires), and objective measurements of sleepiness and sleep quality (polysomnography).

The manuscript should be revised to reflect these limitations. I.e., "Coffee intake shows low association with [subjective estimates] of sleep or daytime sleepiness"
Similarly - Line 166 "Likewise and confirming the validity of this metric...," should be softened to "supporting the validity...." or "supporting the use...."

We understand these concerns and we agree that this should be more clearly stated in our manuscript. Hence, we have gone through and revised our text to better consider the inherent uncertainty in self-reported data.

At this northern latitude, did time of year significantly affect estimated coffee consumption or subjective estimates of sleepiness or sleep quality? Line 189 - Was BMI a better correlate for loud snoring than coffee consumption? Is seasonal sleep and sleepiness and caffeine consumption another uncontrolled variable in interpretation?

This is a very interesting question that we have asked ourselves during the course of our work. It is not entirely inconceivable that the stark differences between the seasons at these northern latitudes, not least regarding the amount of daylight, have a significant impact on individuals' sleepiness and sleeping habits, and consequently on their coffee consumption patterns. However, there is no collected information available in SCAPIS that would enable analysis of this, as participants completed the provided questionnaire continuously over the years that the SCAPIS study was conducted. We have now addressed this potential limitation in the discussion section of our manuscript.

Terms need to be sufficiently defined to eliminate the apparent discrepancy in conclusion of line 183 and line 191. 183 - "This relationship was reversed with increased coffee consumption, where high consumption was associated with better sleep quality..." and line 191 - "there was a slight association between higher coffee consumption and lower overall sleep score." (What is the difference between the terms "sleep quality" and "sleep score." The methods only define a "sleep quality score.") As written, these lines seem to be contradictory.

Thank you for pointing this out. We understand the confusion surrounding the terminology and realize that we have not been sufficiently clear in our reporting. The sleep quality that we refer to on line 183 is the perceived quality of sleep under usual circumstances as self-reported via questionnaire by the participants. On line 191, we refer to the sleep score, which is an aggregated score based on all the seven sleep variables in the study. While it could be argued that this sleep score fills the same function as the self-reported sleep quality, we still think it contributes with an overall estimate of sleep not captured by the self-reported perceived sleep quality alone. Furthermore, just to clarify, lower sleep score means better overall outcome, and therefore line 183 and line 191 does not contradict each other. However, we understand the confusion, and we have therefore updated our manuscript to better explain the difference between these two terms. We now also refer to the “sleep score” instead of “sleep quality score” to avoid confusing it with the participant-reported sleep quality.

Line 201 - "Stress" correlates well with subjective estimates of sleep quality and sleepiness; perhaps the methods section could include more discussion of how this variable was determined.

All variables used in this study are derived from self-reported data collected through questionnaires. As the questionnaire belongs to SCAPIS, we (the authors of the present study) do not have access to, and therefore are not able to share, the questionnaire in its original form as given to the participants. For more information and access to the questionnaire used in SCAPIS, please directly contact SCAPIS at scapis@scapis.org.

Specifically, the variable stress corresponds to subjective experience of stress, obtained through the core SACPIS questionnaire. The question was worded in the following way (translated from Swedish): “By stress we mean feeling tense, irritable, anxious or having sleeping difficulties as a result of conditions at work or at home. Have you experienced this?”. Response options were “Don't want to/can't answer”, “Never experienced stress”, “Experienced some periods of stress”, “Some periods of stress during the last five years”, “Constant stress during the last year”, and “Constant stress during the last five years”.

In our revised supplementary materials, we have now added a table (Table S1) with details on each of the variables included in the study, their original collection and use.

Prior studies suggest slow metabolizers suffer more hyperglycemia, hypertension and MI. Further, the "Sleep quality score" has correlated with cardiovascular events.... I do not appreciate correlation among slow and fast metabolizers and usual sleep complaints associated with cardiovascular risk.

In the light of the lack of clear association between coffee consumption and sleep in our study, the rs762551 stratification analysis was intended as a way to delve a bit deeper into subgroups based on caffeine metabolism. However, due to the very small effect sizes and problems fitting this analysis into the study as a whole, we have decided to remove it. Thus, the revised manuscript now contains three main figures instead of four, which we believe have rendered our reporting more focused and streamlined.

OVERALL, recommend de-emphasize negative results (for sleep and sleepiness) and emphasize novel (GWAS) findings.

Thank you for the thorough review of our work. Regarding the novel GWAS findings please refer to our reply to the reviewer’s first comment.
